# Mortality Reduction Associated with HIV/AIDS Care and Antiretroviral Treatment in Rural Malawi: Evidence from Registers, Coffin Sales and Funerals

**DOI:** 10.1371/journal.pone.0010452

**Published:** 2010-05-04

**Authors:** Beatrice Mwagomba, Rony Zachariah, Moses Massaquoi, Dalitso Misindi, Marcel Manzi, Bester C. Mandere, Marielle Bemelmans, Mit Philips, Kelita Kamoto, Eric J. Schouten, Anthony D. Harries

**Affiliations:** 1 Thyolo District Health Services, Ministry of Health and Population, Thyolo, Malawi; 2 Medical Department (Operational Research), Brussels Operational Center, Medecins sans Frontieres, Luxembourg, Luxembourg; 3 Medecins sans Frontieres, Thyolo, Malawi; 4 Thyolo District Assembly, Thyolo, Malawi; 5 Analysis and Advocacy Unit, Brussels Operational Centre, Médecins sans Frontieres, Brussels, Belgium; 6 Ministry of Health and Population, Lilongwe, Malawi; 7 Management Sciences for Health, Lilongwe, Malawi; 8 International Union against Tuberculosis and Lung Disease, Paris, France; 9 London School of Hygiene and Tropical Medicine, London, United Kingdom; University of Cape Town, South Africa

## Abstract

**Background:**

To report on the trend in all-cause mortality in a rural district of Malawi that has successfully scaled-up HIV/AIDS care including antiretroviral treatment (ART) to its population, through corroborative evidence from a) registered deaths at traditional authorities (TAs), b) coffin sales and c) church funerals.

**Methods and Findings:**

Retrospective study in 5 of 12 TAs (covering approximately 50% of the population) during the period 2000–2007. A total of 210 villages, 24 coffin workshops and 23 churches were included. There were a total of 18,473 registered deaths at TAs, 15781 coffins sold, and 2762 church funerals. Between 2000 and 2007, there was a highly significant linear downward trend in death rates, sale of coffins and church funerals (X^2^ for linear trend: 338.4 *P*<0.0001, 989 *P*<0.0001 and 197, *P*<0.0001 respectively). Using data from TAs as the most reliable source of data on deaths, overall death rate reduction was 37% (95% CI:33–40) for the period. The mean annual incremental death rate reduction was 0.52/1000/year. Death rates decreased over time as the percentage of people living with HIV/AIDS enrolled into care and ART increased. Extrapolating these data to the entire district population, an estimated 10,156 (95% CI: 9786–10259) deaths would have been averted during the 8-year period.

**Conclusions:**

Registered deaths at traditional authorities, the sale of coffins and church funerals showed a significant downward trend over a 8-year period which we believe was associated with the scaling up HIV/AIDS care and ART.

## Introduction

Malawi (population 13 million) is a small resource-limited country in Southern Africa with an estimated HIV prevalence of 12% [1] ranking it among the ten highest HIV prevalence countries worldwide. In 2003, the National AIDS Commission estimated that 900,000 individuals were living with HIV/AIDS and an estimated 170,000 people were in urgent need of antiretroviral treatment (ART). An estimated 90,000 deaths were attributed to HIV/AIDS related disease each year.

Thyolo district in rural southern Malawi with an adult HIV prevalence rate of 21%[1] is one of the pioneer districts in the country to start offering ART. ART was initiated in this district in early 2003 and three years later when it was felt that the implementation model was feasible, efforts were made to ensure district wide access to treatment. In 2006, there were 57,438 people estimated to be living with HIV/AIDS of whom approximately 11,487 were thought to be in urgent need of ART. By December 2007, more than 10,000 individuals had been started on ART with an additional 6,000 patients placed on treatment thereafter. These figures are well in line with the Ministry of Health (MOH) universal access target of providing ART to 80% of those in need of therapy.

With the introduction of ART into sub-Saharan Africa there has been much discussion about how this might reduce HIV-related deaths at a population level[2]–[6]. However, except for a few studies[7]–[9] that have focused on urban areas or for shorter study periods and one study from rural South Africa [10], there is very limited published information from the region on the impact of HIV/AIDS care and ART on mortality in rural settings.

We report on the trends in all-cause mortality in a rural district of Malawi that has successfully scaled-up HIV/AIDS care including ART to its population, over a eight year period (2000–2007) through corroborative evidence from a) registered deaths at the traditional authorities, b) coffin sales and c) church funerals.

## Methods

### Study design

Retrospective cross-sectional descriptive study

### Study setting and population

The study was conducted between February and July 2008 in Thyolo district, on of the largest rural district in Malawi with approximately 592,630 inhabitants in 2007[11]. The study covered the period January 2000 to December 2007. Since 1997, Médecins Sans Frontières (MSF-OCB) has been working in close collaboration with the district health services of the MOH in setting out a comprehensive HIV/AIDS prevention and care program. Chronologically, the project evolved as follows: voluntary counselling, HIV testing and home based care in January 1998; HIV testing for all tuberculosis (TB) patients including cotrimoxazole prophylaxis and management of opportunistic infections in June 1999 [12]; Prevention of Mother to Child Transmission[13] and HIV/AIDS consultations at hospital and health centres in 2002; ART initiation at the district hospital in 2003 which progressively scaled up to include health centres. The treatment protocols have all been approved by the MOH and have been in line with World Health Organization guidelines and Malawi National ART Guidelines[14]. HIV/AIDS care and ART are provided free of charge.

Thyolo district is divided into 12 administrative areas known as “Traditional Authorities and sub-traditional authorities (TA's). Of these, five TAs (4 TAs and 1 sub TA) that are relatively close to Thyolo district hospital (Nchiramwela, Bvumbe, Kapici, Chimaliro and Nanseta) and that were also the first to access HIV/AIDS care and ART were included in the study. Between 50–55% of the total population of Thyolo live in these five TAs.

### Traditional authorities, coffin workshops, churches and other sites as sources of data on population mortality

There is no reliable system that captures population level deaths at a district level in Malawi. There are district health surveys which are conducted every four years, but they are not specifically designed to measure impact indicators. At the health service level deaths are only reported when they involve in-health facility deaths. We thus chose three somewhat parallel sources of data, namely deaths registered at TAs, coffin sales and church funerals.

### Traditional authorities

Each TA comprises several villages with each village having a headman. A group of villages reports hierarchically to a group village headman who then reports to the chief of the traditional authority. Each village headman is responsible for maintaining a register (or often a hard-cover exercise book) for recording deaths and births and these data are transmitted on a monthly basis to the group village headman and then to the central office of the TA. Group village headmen are held accountable by the TA chief for the timely and accurate reporting of deaths. These data have existed for over 25 years in Malawi when they were used as the tax payers roll/register (Kaundula in the local language) before democratic elections in the early 90's. Since a village headman was denied his title if he did not maintain a good register, this culture has been largely revered and maintained. These data are still very relevant to the TA authorities as it is used for assistance subsidies (fertilizers, seed grain) for households, prevention of property and land grabbing by neighbours and family relatives and for planning. Data are cross checked by the TA central office staff through site visits and verification of registers where relevant. This source of data is thus the single most reliable and available measure of population-level deaths. A formal letter was solicited from the district commissioner in order to facilitate contact and administrative procedures linked to review of death registers at each of the TA central administration offices. In cases where data were missing or unclear at the TA, visits were made by the study team to the group village headmen or villages and data were physically verified through their registers.

### Coffin workshops

Coffin workshops exist in each traditional authority, and it is general practice in Thyolo to bury people in coffins. Although we used coffin sales at known coffin workshops as a proxy for deaths, it is likely that underreporting occurs since coffin sales are influenced by varying coffin prices and household affordability. People may also resort to cloth burials (a common practice during the study period as many households could not afford coffins) or construction of coffins at home [15]. All coffin workshops in the 5 TA's were visited and the owner(s) were informed of the objectives of the study. The “Coffin register book” or “receipt book” for the period 2000–2007 was used to collate information on total numbers of coffins sold during each year. Particular attention was paid in the initial discussions with coffin workshops to clarify that the purpose of the study was “purely medical”, to allay potential fears that the enquiries might be linked in any way to revenue/taxing authorities and to assure that the data collected would not have identifiers.

### Church funerals

Conducting funeral services at churches is common as Thyolo is a predominately Christian district. All funerals include a fee and are thus registered at the church for accounting purposes. Church funeral registers were thus used to gather data on the total number of registered church members and registered funerals among them. However, as a proxy for deaths, it is likely that underreporting occurs as not all households might opt for, nor can afford, formal church funerals.

Other potential data sources explored during this study included tea and tobacco estate authorities for registered deaths among employees. However, data could either not be collected at these sites or was of poor quality and therefore data from this source were not included in the analysis.

### Statistical analysis

Data from TAs, coffin workshops and churches were aggregated by year and standardised per 1000 inhabitants in the five TAs (for deaths and coffin sales) and per 1000 members for church funerals. Yearly population data were based on Demographic Health Surveys (DHS), census data and sentinel surveillance data[11] DHS are done every 4 years and the district population for that year is based on the DHS results. In-between the DHS surveys the district traditional authorities conduct their own household census and, these data serves as the population figure.

The total numbers of individuals ever enrolled in HIV/AIDS care and ART by year were derived from the Thyolo-MSF FUCHIA data base (FUCHIA, Epicentre, Paris, France.) and routine reporting system. X^2^ test for trend was used to test for linear trends. The level of significance was set at *P* = 0.05 or less, and 95% confidence intervals (CI) were used throughout. Data were analysed using the STATA 8.0 software (Stata corporation, Texas 77845, USA).

## Results

### Summary of sites used for data collection

A total of 210 villages (including 210 village headmen and 27 group headmen), 24 coffin workshops and 23 churches were included in the inventory for data collection in the 5 TAs. Of these; data was missing from:- one group headman (misplaced the register for data covering the entire study period); 17(8%) village headman (who did not have registers covering data for the entire study period) and 10 villages had no information on deaths for the year 2000; 2 coffin workshops that were unable to show coffin registers or cash receipts; and one coffin workshop owner refused to participate.

### Registered deaths at traditional authorities and relation to enrolment in HIV/AIDS care and ART

**Figure 1:**
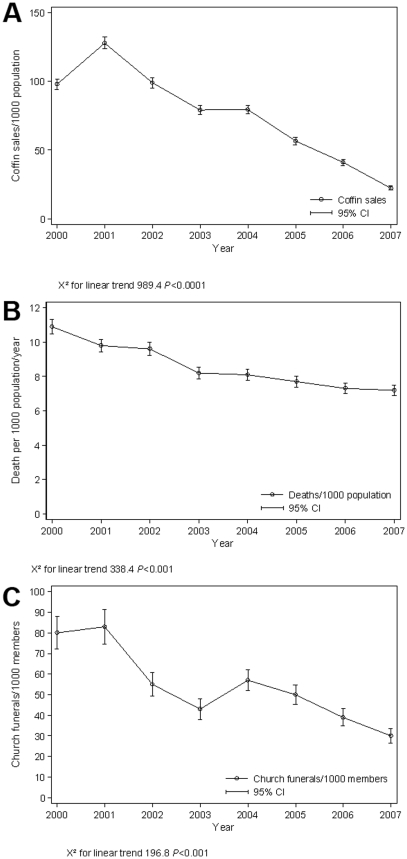
Trends in registered deaths, coffin sales and church funerals. **1a**) Trends in registered deaths at traditional authorities, (2000–2007) Thyolo, District, Malawi. **1b**) Trend in coffin sales (2000–2007), Thyolo, District, Malawi. **1c**). Trend in registered church funerals (2000–2007), Thyolo, Malawi.

Over the study period (2000–2007), there was a total of 18,473 registered deaths at the 5 TAs. **Table 1** shows (by year) the population in the five TAs, enrolment in HIV/AIDS care, placement on ART and the trend in all-cause mortality. This shows that death rates reduced with time as the percentage of people living with HIV/AIDS (WHO stage 3 & 4 or with CD4 counts <250 cells/mm^3^) enrolled into care and ART increased. There is a highly significant linear trend in reduction of deaths between 2000 and 2007(X^2^ for linear trend:338 *P*<0.0001, **Figure 1a**). Using data from TAs as the most reliable source of data on deaths, death rate reduction was 3.4/1000 for the period (Overall death rate reduction  = 37%, 95% Confidence Interval, CI:33–40). The mean annual incremental mortality rate reduction was 0.52/1000/year. Extrapolating these data to the entire population of Thyolo, there would have been an estimated 10,156 (95% CI:9786–10259) averted deaths during the period. **(** **Table 2** **)**

**Table 1:** Registered deaths at five traditional authorities and relationship to enrolment in HIV/AIDS care and antiretroviral treatment (2000–2007), Thyolo district, Malawi.

Year	Total population	Total registered deaths	Death rate/1000 population (95% CI)^a^^,^ b	Number ever enrolled in HIV/AIDS care^c^^,^ d	Number ever started on ART^e^
**2000**	226507	2457	10.9 (10.4–11.2)	770	0
**2001**	242081	2364	9.8 (9.4–10.2)	1484	0
**2002**	248918	2407	9.6 (9.3–10.1)	2711	0
**2003**	278891	2299	8.2 (7.7–8.4)	4383	421
**2004**	281849	2305	8.1 (7.8–8.5)	6609	1550
**2005**	288228	2226	7.7 (7.0–7.6)	8765	3145
**2006**	295739	2167	7.3 (7.0–7.6)	11622	6216
**2007**	310522	2248	7.2 (6.9–7.5)	14627	10215

a. 95% Confidence Interval.

b. X^2^ for linear trend  = 338.4 *P*<0.001.

c. Enrollment in HIV/AIDS care applies to individuals in World Health Organisation clinical stage 3 and 4 or with CD4 counts <250 cells/mm^3^ irrespective of clinical stage.

d. HIV/AIDS care implies management of opportunistic infections, cotrimoxazole preventive prophylaxis and nutritional support.

e. ART- Antiretroviral treatment.

**Table 2:** Death rates from 5 traditional authorities projected to estimate averted deaths in the entire Thyolo district, Malawi.

Year	Total Population Thyolo district (All Tas)	Death rates/1000 population a (95% CI)	Estimated number of deaths^b^ (95% CI)	Projected number of deaths^c^ (95% CI)	Estimated number of lives saved (95% CI)
**2000**	478494	10.9 (10.4–11.2)	5215 (4976–5359)	5215 (4976–5359)	_
**2001**	492495	9.8 (9.4–10.2)	4826 (4629–5023)	5368 (5122–5516)	542
**2002**	507426	9.6 (9.3–10.1)	4871 (4719–5125)	5531 (5277–5683)	660
**2003**	523162	8.2 (7.7–8.4)	4289 (4028–4394)	5702 (5441–5859)	1413
**2004**	539610	8.1 (7.8–8.5)	4371 (4209–4587)	5881 (5611–6064)	1510
**2005**	556700	7.7 (7.0–7.6)	4287 (3896–4231)	6068 (5789–6235)	1781
**2006**	574384	7.3 (7.0–7.6)	4193 (4021–4365)	6260 (5974–6433)	2067
**2007**	592630	7.2 (6.9–7.5)	4266 (4089–4445)	6459 (6163–6637)	2193
**TOTAL**	-	-	**36318** (34567–37529)	**46484** (44353–47786)	**10156** (9786–10257)

TAs, Traditional authorities; CI, Confidence Interval.

a. Death rate from registered deaths at traditional authorities in 5 TAs.

b. Number of deaths extrapolated by year for the entire Thyolo District assuming the same rates of death per year as in the 5 TAs.

c. Number of projected deaths assuming a death rate of 10,9 (95% CI:10.4–11.2)/1000/year that was observed in 2000 in the 5 TAs.

### Trends in coffin sales at coffin workshops

A total of 15,781 coffins were sold in the 5 TAs during the study period (**Table 3**). There is a highly significant downward linear trend in the rate of coffin sales/1000 inhabitants over the period (X^2^ for linear trend 989 *P*<0.0001, (**Figure 1b** **.**)). The total number of coffins sold (15781) is 13% less than the total number of registered deaths in TAs (18,081). This difference is likely explained by cloth burials and home made coffins which are not reported by coffin workshops. An increase in coffin sales in 2001 is explained by the fact that some tea and coffee estates in the district started paying for coffins for their employees and their families, but this was felt to be unsustainable and thus stopped at the end of 2001.

**Table 3:** Coffin sales (2000–2007) at coffin workshops in 5 traditional authorities in Thyolo district, Malawi.

Year	Total population	Total number of coffins sold	Coffin sale rate/1000 population^a^ (95% CI^b^)
2000	226507	2219	98 (94–102)
2001	242081	3103	128 (124–132)
2002	248918	2467	99 (95–103)
2003	278891	2210	79 (75–83)
2004	281849	2240	80 (76–83)
2005	288228	1632	57 (54–59)
2006	295739	1216	41 (39–44)
2007	310522	694	22 (21–24)

a. X^2^ for linear trend  = 989.4, *P*<0.0001.

b. 95% Confidence Intervals.

### Trends in registered church funerals

A total of 2762 church funerals were conducted in the 23 churches (**Table 4**) There is a highly significant downward linear trend in the rate of church funerals over time (X^2^ for linear trend 197, *P*<0.0001, **Figure 1c** **.**)

**Table 4:** Registered church funerals in the 5 traditional authorities (2000–2007), Thyolo district, Malawi.

Year	Number of registered church members	Total number of church funerals	Funeral rate/1000 church members^a^ (95% CI^b^)
**2000**	40699	324	8.0 (7.1–8.8)
**2001**	42701	354	8.3 (7.4–9.1)
**2002**	60377	330	5.5 (4.8–6.1)
**2003**	64131	277	4.3 (3.8–4.8)
**2004**	80694	463	5.7 (5.2–6.3)
**2005**	82083	411	5.0 (4.5–5.5)
**2006**	83631	340	3.9 (3.5–4.3)
**2007**	86986	263	3.0 (2.6–3.4)

a. X^2^ for linear trend  = 196.8, *P*<0.0001.

b. 95% Confidence Intervals.

## Discussion

In a rural district of Malawi that has embarked on scaling up HIV/AIDS care and ART, there appears to be a very significant linear trend in reduction of all-cause mortality at population level. This is also one of the first reports to use proxy indicators of death such as coffin sales and church funerals both of which show a declining trend over time.

Malawi like other high HIV-prevalence countries in sub-Saharan Africa, has been desperately trying to scale-up HIV/AIDS care and ART as a lifesaving intervention to its population. In this light, the finding of an overall 37% reduction in all-cause mortality at population level is very encouraging. Similar reductions in population level mortality have been reported from Northern Malawi [9], Addis Ababa[7], Botswana[16] and rural South Africa [10]. In Botswana, the national program estimated that the annual number of AIDS related deaths halved from a peak of 15,500 in 2003 to 7400 in 2008 (7100 averted deaths) with an 80% treatment coverage[16]. These reductions are however less dramatic and less rapid than those reported from New York [17] and Sau Paulo[18] (63% in 2 years, 65% in 7 years respectively). The reasons for the slower decline in population deaths in our setting (like other African countries[7], [9], [10], [16] could include the fact that a) the epidemic peaked earlier compared to the timing of ART roll out, b) the number of AIDS cases are far larger resulting in difficulties for the relatively weak health infrastructure to provide universal and comprehensive HIV/AIDS care c) socio-cultural and economic factors impede access and demand for care and d) the fact that patients present late for ART which compromises their survival even when on the life-saving medication[19].

Although we cannot know with certainty that the observed reduction in deaths is directly linked to HIV/AIDS care and ART roll out, the evidence is however highly suggestive and intuitive [7]–[9]. In a high HIV prevalence country like Malawi, up to 65% (the great majority) of adult deaths can be attributed to HIV[9]. A recent study from South Africa [10] where the cause of death was known showed that reductions in all-cause mortality were largely attributed to specific reductions in HIV related deaths among people on ART.

Reducing HIV/AIDS related deaths through specific interventions should therefore inevitably have an impact on all-cause mortality. This is also evident from a recent ecological analysis of all cause mortality and HIV treatment [20] which demonstrated that when large numbers of people living with HIV receive care and ART in a mature epidemic, the mortality rate or the increase in the rate will decline. Although we do not have specific data to support this, we do not think that there were other factors that could have confounded the results like a significant improvement in social conditions such as employment opportunities or earning capacity of inhabitants. In fact, the prices of petrol, diesel and paraffin have generally shown an upward trend over the study period with a consequent increase in consumer prices [21]. These are more likely to negate the overall impact of HIV/AIDS interventions on all-cause mortality. In any case, improvements in socio-economic factors alone, without access to HIV/AIDS treatment are unlikely to have any significant impact in a mature HIV/AIDS epidemic. There were reports of food shortages in 2001 and eventually a famine in 2002[22]. Although we should have expected to see an increase in deaths during this period, the downward trend in deaths was sustained.

There were also no specific epidemics resulting in exceptional mortality during any particular year of the study period and immunisation coverage in Malawi is generally high and constant [23].

Although it was only in 2007 that the target of universal access (80% of those estimated to be in urgent need of ART receive treatment) was achieved in Thyolo, mortality reduction was evident prior to this year with the largest annual declines, earlier in the study period. This is probably explained by the fact that mortality is influenced not just by ART alone, but also by other HIV/AIDS related care interventions such as cotrimoxazole prophylaxis [12], availability of drugs for prompt management of opportunistic infections, nutritional support and the presence of community support groups and networks[24]. This package existed prior to 2004 when ART initiation was started in our setting. Furthermore, MSF started its district level support in 1997 and in particular included, drug support to all peripheral health facilities for three key diseases namely malaria, respiratory disease and diarrhoea as from the year 2000. These interventions might well have contributed to reduction of death rates particularly child mortality in the earlier years. However, this would have had a relatively rapid impact which would have subsequently remained constant as MSF sustained drug support for these diseases during the entire study period (2000–2007). In any case, such interventions in their own right might at best delay, but not prevent mortality in a mature epidemic like in Thyolo where patients need ART.

The strengths of this study are that about half of the total population of a district with half a million inhabitants was included, and deaths were reliably verified through registers and are thus likely to reflect the reality on the ground. We also successfully managed to use three innovative proxy indicators of death in the absence of a formal and functional vital registration system. The limitations of the study are that:- i) it is based on simple observational data with the usual shortcomings; ii) we had missing data from 8% of villages and data on deaths are thus underestimated. However, since these data were missing for the entire study period, they do not influence the trend in mortality; iii) ten villages had no information on deaths for the year 2000. As this is the year of study start, this would have rather negated the impact of ART over the subsequent years and not the contrary; iv) importantly our data were not stratified by age and sex as this information was inconsistently recorded. The concept of age in traditional communities like Thyolo is anyway relative and its accuracy is in doubt. However, lack of age specific mortality data implies that interventions that significantly reduce child mortality will impact on all-cause mortality. Since vaccination coverage was high and specific support for malaria, diarrhoea and respiratory infections were sustained during the entire period this effect should have remained constant. In light of these potential limitations, we have kept the general focus on “trend analysis” of all-cause (crude) mortality so that age-specific and sex-specific mortality as well as any under-reporting bias remains stable during the study period. The reporting is also focused on mean mortality reduction over time; v) finally, we did not have data on the cause of mortality as post-mortems are not done in this setting.

In addition to the reduction in deaths, this study highlights two important additional issues that merit discussion. First, most African countries do not record vital registration data[25] and where it exists this is limited to health facility based data. However, the latter is not representative as more than half of all deaths that occur do so outside of health facilities and this is typical of other rural African settings[26]–[27]. In the absence of a viable vital registration system or reliable mortality statistics from the health information system, AIDS mortality estimates have been dependent on mathematical models[28] which could be quite different from the reality on the ground[26]. In this light, the existing death registration system at the TAs could provide an opportunity to fill this gap.

Second, this system runs parallel to the health management information system (HMIS) and at the moment remains solely for use by TAs. We need to explore whether the TA registration system can be enhanced and linked up with the HMIS to provide timely population level age- and sex-related mortality data that would assist also with child and maternal mortality data. This is particularly pertinent for assessing the impact of national programs and particularly as we approach 2015, reporting against targets in the Millennium Development Goals 4 and 5. Dialogue to explore possible ways to facilitate information transfer (e.g. a system for monthly or even trimestrial data transmission) between the TA systems and HMIS should be explored. This issue would benefit from further evaluation, training and support where necessary as it provides an important potential opportunity to improve information on vital data and reliably assess the impact of health and other interventions at population level.

In a rural district of Malawi that has scaled up HIV/AIDS care and ART, corroborative evidence from registered deaths at the traditional authorities, coffin sales and church funerals show encouraging evidence of a significant downward trend in reduction of population level deaths.

### Ethical approval/statement

General measures are employed in all Thyolo district health facilities to ensure patient confidentiality, consent for HIV testing, and counselling and support for those who receive a positive HIV test result. The data in this study did not include patient identifiers. This study was formally approved by the District Commissioner of Thyolo who is also head of the Assembly of Traditional authorities. The Malawi National Health Science Research Committee provides general oversight and approval for the collection and use of data for monitoring and evaluation purposes, and approved this study. The study also received ethical approval from Ethics Review Board of the International Union Against TB and Lung Disease, Paris.
